# Impact of Horse Grazing on Floristic Diversity in Mediterranean Small Standing-Water Ecosystems (SWEs)

**DOI:** 10.3390/plants11121597

**Published:** 2022-06-17

**Authors:** Giuseppe Fenu, Arianna Melis, Maria Silvia Pinna, Maria Cecilia Loi, Giulia Calderisi, Donatella Cogoni

**Affiliations:** Department of Life and Environmental Sciences, University of Cagliari, Via Sant’Ignazio da Laconi, 13, 09123 Cagliari, Italy; gfenu@unica.it (G.F.); melisarianna17@gmail.com (A.M.); loimc@unica.it (M.C.L.); giulia.calderisi@gmail.com (G.C.); d.cogoni@unica.it (D.C.)

**Keywords:** discriminant plants, diversity index, floristic richness, horse grazing, Giara di Gesturi, Sardinia, Special Areas of Conservation

## Abstract

*Small standing-Water Ecosystems* (SWEs), despite their pivotal ecological role due to their participation in hydrogeological processes and their richness in biodiversity, seem to be often overlooked by the scientific community. In this study, the vascular plant diversity in some representative SWEs, that host a peculiar assemblage of plant and animal species, was investigated in relation to the disturbance effects of a wild horse population. A total of 50 plots, equally distributed in small and large SWEs, were surveyed and a level of disturbance was attributed to each plot. We found greater species richness in small and undisturbed SWEs, which suggests the negative impact of horse grazing on the richness of plant species in this type of habitat. Significant differences in plant assemblage were found according to the disturbance level, whereas, contrary to what was observed for species richness, no differences were detected based on their size. The diversity indices, used to evaluate the richness and diversity in these areas, recorded the highest values for small and undisturbed areas. This result highlights that the disturbance of the horse grazing plays a pivotal role in affecting the diversity and richness of species in the SWEs. These findings suggest that SWE systems should be analyzed considering these areas as unique in order to allow the conservation of the plant richness and biodiversity of the SWE systems in conjunction with the protection of horses.

## 1. Introduction

Small standing-water ecosystems (SWEs) are shallow, lentic water bodies with frequently small surface areas. Their share is estimated at 30% of the standing water surface of the Earth [1,2,3] and, in the Mediterranean Basin, SWEs can be either perennial or seasonal, with a complex pattern of dynamic phases that create a peculiar ecosystem [4]. This definition of SWEs, therefore, encompasses a wide range of ecological situations, including the typical Mediterranean temporary ponds [5,6].

Due to their natural heterogeneity, SWEs play a pivotal ecological role. First, they participate in hydrological and biogeochemical processes [2,7]. Second, they are considered a biodiversity hotspot, being an incredibly wide habitat with high species diversity [6,8]. However, due to their ephemeral nature and ecological features, SWEs are particularly vulnerable and threatened ecosystems, both directly and indirectly, by anthropogenic-related activities such as the intensive exploitation of lands, eutrophication, pollution, the introduction of alien species, and climate change [4,9,10,11,12,13]. Although, the specialist species of SWEs show remarkable growth in terms of cover in the wettest period, the sensitivity to drought may make these species particularly vulnerable to climate change in the Mediterranean region. Such phenomenon may cause irreparable damage to the hydrologic cycle of Mediterranean SWEs [13,14,15,16]. Nevertheless, some studies highlighted that the intensity and frequency of the disturbances generated by alternative phases of flooding and dry stages allow the presence and stable growth of plant species in many Mediterranean SWEs [17,18], indicating a certain resilience to these changes [10,19]. The interaction of all these threats has a general negative effect on freshwater habitats [10], and on the specialized vascular flora that colonizes SWEs. Although SWE are globally abundant and their ecological importance is unquestionable, there is a general lack of studies on these habitats, which are often neglected and overlooked by scientific communities and, consequently, in conservation policies [20].

In Europe, the conservation of freshwater ecosystems is one of the purposes of the 92/43/EEC “Habitats” Directive, which designates and includes several freshwater habitats in Annex 1; in this directive, SWEs are included in the “standing water group”, a group that also involves a particular type of habitat of priority community interest called “Mediterranean temporary ponds” [6]. Furthermore, under the Habitats Directive (Art. 3 and 4), Member States select Special Areas of Conservation (SACs) to ensure the favorable conservation status of each habitat type and species throughout their range in the EU. These sites become an integral part of the Natura 2000 network (http://ec.europa.eu/environment/nature/natura2000/; accessed on 10 March 2022).

The need for an accurate and scientifically based management strategy becomes indispensable when, in the same site, several species of conservation interest coexist and interact in a potentially negative way with each other or peculiar habitats. In these situations, where the overriding interest should be to preserve the ecological balance of the system in all its components, management should also consider containment measures for a species if this represents damage to other species or habitats. Therefore, increased knowledge of flora and vegetation is vital to establish sound management activities in SWEs to conserve their natural heritage.

In this framework, a controversial topic concerning these ecosystems is the impact of livestock grazing and trampling, which can play a positive, neutral, or negative effect [21,22,23,24,25,26]. In fact, many studies assessed both positive and negative effects of livestock on plant species and communities, suggesting a complex network of factors implied in plant species’ response to stress [27].

Grazing threatens plant species directly, damaging plant tissues through nutrition, and indirectly as a result of trampling, consumption, water quality degradation, and deterioration in soil conditions. Alternatively, indirect impacts of livestock grazing can sometimes result in the increase in the number of palatable individuals and juvenile plants [27,28], or benefit the survival or recovery of plant species by controlling alien plants, reducing thatch, maintaining hydrologic functions, creating some bare ground, and increasing plant diversity [27]. The complex interaction among all these factors suggests the necessity for a specific grazing management strategy as a tool to protect environmentally valuable habitats [27,29,30].

One of the most common management measures used to tackle plant species and habitats from grazing is the erection of fences, which is also used for SWEs. Fencing has positive effect by preventing plant species from being grazed and trampled by herbivores [24,31]; conversely, over medium- or long-term periods, fencing produces important changes in vegetation composition and structure [32]. Nevertheless, the results of assessments of fencing outcome are contrasting, depending on species, and habitat or microhabitat; hence, fencing needs a case-by-case evaluation of its suitability and the most favorable option [24].

The basaltic plateau of “Giara di Gesturi” (C-Sardinia), an area of high naturalistic interest, is unique because it hosts a high density of SWEs, mainly consisting of temporary ponds and wetlands. These are characterized by a wet period (October–June) in which SWEs are filled with rainwater that is not absorbed by the basaltic ground, and a dry period (June–October) in which the smaller pools are completely dried, and the larger pools experience a significant decrease in the water level [33]. These freshwater ecosystems are characterized by a peculiar assemblage of plant and animal species, included a population of small wild horses. The main impact detected at the site is the grazing by ungulates, in particular cattle and goats, in addition to wild horses. Although the positive effect of traditional grazing on plant diversity is documented, even for Mediterranean SWEs [34], the impact of horses grazing has received little scientific attention; in particular, on the basis of our knowledge, this issue has never been investigated in our study area. Nevertheless, studies focusing on horse grazing have shown conflicting results regarding the effects of this practice on plant community diversity. For example, recent research on grassland vegetation highlighted the potential of horse grazing to maintain species richness [35]. Barry and Huntsinger [27] reported a benefit for plant species from grazing, which allowed habitat maintenance and improvement in SWEs by the control of alien plants, and the positive effects of horse grazing on nature conservation benefits have been demonstrated [36,37]. Similarly, in a study on wetlands by Marion et al. [38], horses had a positive impact on species richness and diversity due to the grazing-induced patchiness. On the contrary, horse grazing has been shown to have negative effects due to high trampling pressure, in addition to negatively influencing vegetation in semi-arid ecosystems [38,39].

The main goal of our study was to investigate the vascular plant diversity in some SWEs and to analyze the effect of disturbance on this diversity; in addition, specific aims of this study were to: (1) quantify the floristic diversity of SWEs of different sizes; (2) determine the effects of disturbance associated with the presence of wild horses on this floristic richness; and (3) suggest management measures that preserve the ecological balance of the SWE system by reconciling the presence of wild horses and conservation of the floristic heritage.

## 2. Results

Overall, 42 different *taxa* were found across the investigated plots. Most of the surveyed *taxa* were therophytes (i.e., 19, representing 45.24% of the total), of which almost all are specialists of SWEs (15 *taxa*). However, the main ecological form was represented by generalist plants of wet habitats (17 *taxa*, corresponding to 40.48% of the total). The species composition appeared to be different according to both SWEs’ size and disturbance level.

In small SWEs, a total of 37 *taxa* were found, with an average of 14 *taxa* per plot, more than double those of large SWEs (6 *taxa*). Species richness was higher in undisturbed areas (14 *taxa*) and lower in disturbed and over-disturbed area (6 *taxa*).

The most frequent species across the whole studied area were *Baldellia ranunculoides*, *Helosciadium crassipes*, and *Eleocharis palustris*; the first of these, *Baldellia ranunculoides*, was assessed in almost all of the surveyed area, with higher cover values in the small and undisturbed SWEs. *Helosciadium crassipes* and *Eleocharis palustris* were more likely found in small and undisturbed areas. However, *Helosciadium crassipes* was quantitatively the preponderant species in disturbed plots.

**Figure 1:**
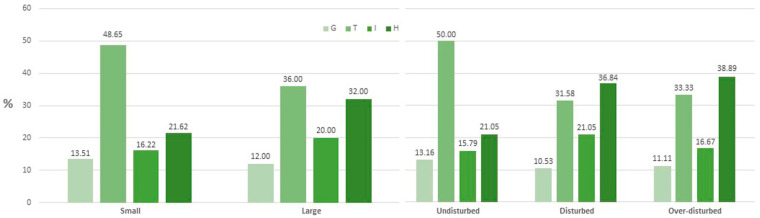
Percentage cover of geophytes (G), therophytes (T), hydrophytes (I), and hemicryptophytes (H) in small and large SWEs (**left**) and in undisturbed, disturbed, and over-disturbed plots (**right**).

The main biological form in both large and small SWEs was therophytes (representing 45.24% of *taxa* overall, i.e., 48.6% in small and 36% in large SWEs) followed by hemicryptophytes (overall 26.19%, i.e., 21.6% in small and 32% in large SWEs); however, in large SWEs, hemicryptophyte reached values comparable to those of therophytes (36%); geophytes and hydrophytes showed a similar pattern in both large and small SWEs (Figure 1 left). A different pattern emerged from the level of disturbance, since, in undisturbed areas, the cover percentage of therophytes reached 50% of the total, whereas, in disturbed and over-disturbed areas, hemicryptophytes dominated (36.8% and 38.9%, respectively), slightly exceeding the value of therophytes (31.6% and 33.3%) (Figure 1 right).

Chorologically, the Mediterranean type dominated (52.4%), but a relevant presence of Cosmopolite and Subcosmopolite species (23.8%) was also found. Among the Mediterranean plant species, the most relevant types were Euri-Mediterranean (14.3%) and Mediterranean-Atlantic (9.5%) species.

**Figure 2:**
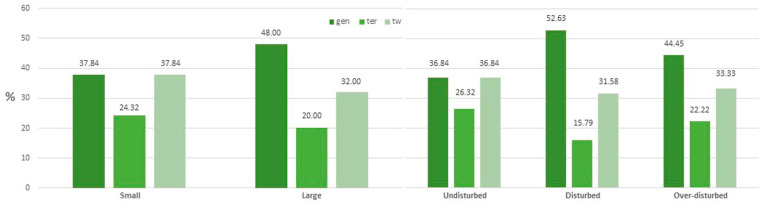
Percentage cover of generalist species of wet habitats (gen), opportunistic terrestrial species (ter), and specialist species of temporary pond (tw) in small and large SWEs (**left**), and in undisturbed, disturbed, and over-disturbed plots (**right**).

Ecologically, generalist species dominated in large SWEs, followed by specialist species, whereas, in small SWEs, generalist and specialist species achieved the same average coverage value (Figure 2 left). Generalist species coverage increased in disturbed plots but subsequently decreased with further increases in disturbance, and always remained higher than in the undisturbed areas (Figure 2 right). Interestingly, the cover percentage of the three categories was similar in small SWEs and undisturbed SWEs.

**Figure 3:**
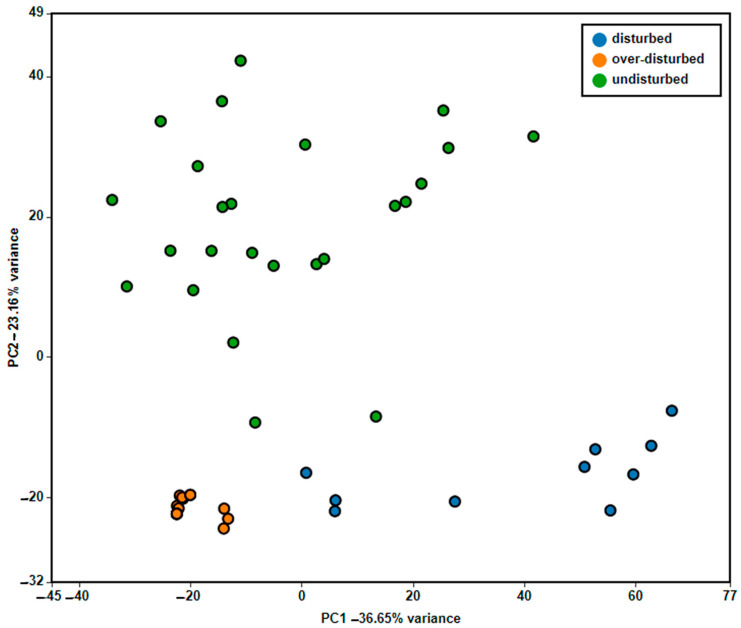
Principal component analysis (PCA) diagram of the vascular plant assemblage related to the disturbance levels in the study area.

The results from PERMANOVA showed significant differences in plant assemblage composition for the *a posteriori* comparisons (*p* < 0.001) according to the disturbance level, whereas no differences based on the SWE size were found; the interaction between SWE size and disturbance level was also statistically not significant (*p* > 0.05). The PCA clearly highlighted the results of this analysis; the first two principal coordinates of PCA explained 36.65% and 23.16% of the floristic variance (Figure 3).

The SIMPER analysis highlighted five discriminant *taxa* in groups of plots sorted by size and treatment (Table 1); SIMPER results revealed a clear separation based on SWEs’ size, indicating a preponderance of *Eudianthe laeta* and *Eleocharis palustris* in small SWEs, and *Alisma plantago-aquatica* and *Illecebrum verticillatum* in large SWEs. The presence of *Helosciadium crassipes* was strong in all SWEs; nevertheless, it reached the highest mean cover values in large SWEs (Table 1). Considering the disturbance level, SIMPER highlighted three species as discriminant in undisturbed SWEs (*Eudianthe laeta, Eleocharis palustris*, and *Helosciadium crassipes*), one in disturbed SWEs (*Helosciadium crassipes*) and two in over-disturbed SWEs (*Alisma plantago-aquatica* and *Illecebrum verticillatum*) (Table 1 and Table 2). The cover values were higher in undisturbed and disturbed SWEs, where the discriminants reached 80%, whereas, in over-disturbed SWEs, the coverage reached a maximum of 20%. SIMPER analysis identified five plant species among assemblages that were discriminated most among years (Table 1 and Table 2). The percentage cover of each species was generally low.

### Diversity Indices

The mean floristic richness was higher in the small and in the undisturbed SWEs, whereas it was lower in large, disturbed and over-disturbed SWEs, with a minimal difference between disturbed and over-disturbed SWEs (Table 3); accordingly, the H_modified_ diversity index showed the same trend but, in this case, the difference between disturbed and over-disturbed SWEs was also significant (Table 3). Conversely, the evenness (E_modified_) index indicated an inverse trend, presenting the lowest mean values in small, undisturbed and disturbed SWEs.

As no alien and endemic plant species were detected, the naturalness index (N) and the endemicity index (EI) were equal to 1 and zero, respectively.

The Kolmogorov–Smirnov test showed significant differences (*p* < 0.001) between small and large SWEs for all indices considered (Table 4). The Kruskal–Wallis One way Analysis of Variance on Ranks, followed by all pairwise multiple comparison tests, indicated that there were significant differences in the number of species depending on the disturbance level; in fact, the number of species in the undisturbed SWEs was statistically higher than that of the disturbed and over-disturbed SWEs, whereas there were no differences between these last two categories (Table 5). The H_modified_ index showed a similar trend, whereas the E_modified_ index showed a different trend, highlighting significant differences between undisturbed and disturbed SWEs compared to over-disturbed SWEs (Table 5).

## 3. Discussion

In this study, the floristic diversity of SWEs and the impact of the disturbance associated with the presence of wild horses on floristic diversity in small standing-water ecosystems was analyzed. Although some studies indicated a positive effect of traditional grazing on plant diversity [27,35,40] and species richness [18] in Mediterranean SWEs, in this study, a greater species richness was found in small and undisturbed SWEs, and the same pattern was also recorded for mean values of floristic richness. This result suggests horse grazing has a negative impact on the richness of plant species in this type of habitat. This finding highlights that livestock grazing is a threat to plant species due to its negative effects, including damage from nutrition or trampling.

Concerning the plant assemblage of the SWEs, significant differences according to the disturbance level were found, whereas, contrary to what was observed for species richness, no differences were detected based on their size. In Mediterranean SWEs, different plant assemblages have been instead observed, associated with an arrangement in three concentric belts, i.e., a central belt, an intermediate belt, and an outer or peripheral belt [6,34,41,42]. This distribution was not observed in our study, since our plots were all concentrated in the same ecological situation, in completely submerged areas with low water depth, and surveyed in June at the beginning of the dry period, which coincides with the SWEs’ peripheral belt.

Overall, the main biological form recorded in both large and small SWEs was therophytes, in agreement with previous studies [43]. In these environments, in fact, the cover of therophytes is linked to their very short life cycles, which are considered an adaptation to survive fluctuations in water availability [18,44,45]. Conversely, differences in coverage of biological form were found based on the level of disturbance; in undisturbed areas, the therophytes dominated, whereas, in disturbed and over-disturbed situations, hemicryptophytes reached the main cover percentage, slightly exceeding the value of therophytes. This result is interesting, as in the Mediterranean area, the geophytes usually become dominant as the disturbance increases, especially that linked to overgrazing [46,47,48]. Surprisingly, our results indicate a progressive decrease in geophytes with increasing disturbance, and this pattern can be linked to the peculiar ecological conditions of the SWEs.

Generalist species are the ecological types that dominated in large SWEs, whereas, in small and undisturbed SWEs, generalist and specialist species achieved the same average coverage values. This finding differs from that reported in Caria et al. [18], which showed that specialist species had the highest overall cover, in addition to being significantly higher in disturbed than in not-disturbed areas. It is noteworthy that, despite such differences in the coverage, specialist species recorded similar values in both different sizes and treatments of SWEs. Consistent with results achieved by Deil [49] and Caria et al. [18], such relative stability of specialist species seems to be related to their adaptation (short life cycle, large perennial seedbanks, phenotypic plasticity) to the harsh conditions encountered in the temporary ponds. A similar trend in cover percentage was detected for the three ecological types of species in small and in undisturbed SWEs. In particular, generalist species coverage was higher in both disturbed and over-disturbed plots, although generalists’ coverage decreased with a further increase in disturbance. This pattern may be linked to the effects of grazing based on its degree of disturbance. Therefore, in disturbed areas, grazing seems to mainly favor the growth of generalist species, whereas in over-disturbed areas characterized by overgrazing, the increase in generalist species appears to be limited by the increase in opportunistic species.

Overall, only five discriminant *taxa* were found, which revealed clear separation according to SWEs’ size and treatment. Some of these species have been already listed and detected in studies focused on flora and vegetation of Sardinian SWEs [18,42,50]. In particular, the specialist *Eudianthe laeta* was predominant in undisturbed SWEs, converse to the findings of Caria et al. [18], in which this *taxon* had higher cover in areas disturbed by wild boars. Instead, *Helosciadium crassipes*, a perennial specialist species, reached its maximum cover in large and disturbed SWEs. This plant requires a more extended submersion period for its vegetative growth than annual species [50]. Such a strategy may explain its high abundance in large SWEs, which are characterized by significant water depth. Furthermore, the higher cover of *Helosciadium crassipes* in the disturbed areas agrees with the results obtained for this species by Caria et al. [18]. It is interesting to highlight that *Illecebrum verticillatum*, an opportunistic species among those preponderant in large and over-disturbed SWEs, was instead listed as a rare specialist *taxon* in Sardinia temporary wet habitats by Bagella et al. [42].

Since diversity indices are often used to evaluate the richness and diversity in different ecosystems, including Mediterranean temporary ponds [42], to evaluate the overall plant species diversity in SWEs, the H_modified_ index was calculated, using a modified index already widely used in very heterogeneous environments [51,52,53]. The H_modified_ index confirmed the same pattern observed for mean and total species richness, with the highest values recorded for small and undisturbed areas. In contrast, in the study carried out by Marion et al. [38] in wet grasslands, horse grazing had a positive impact in terms of species richness and the Shannon diversity index. Moreover, H_modified_ index values calculated in the undisturbed SWEs were statistically higher than those recorded in the disturbed and over-disturbed SWEs, whereas no statistical differences were found between the last two categories of disturbance. This latter result seems to highlight that the presence of disturbance, i.e., horse grazing, compared to the level of the disturbance itself, plays a pivotal role in affecting the diversity and richness of species in the SWEs.

The evenness (E_modified_) index highlighted an opposite trend compared to H_modified_, presenting instead the lowest values in small, undisturbed and disturbed SWEs. These results indicate that, in these areas, there was less homogeneity of species, i.e., a species that dominates the others, compared to large and over-disturbed areas. This is probably the result of two main drivers, namely, the selective effect of horse grazing combined with the greater resistance of some plant species than others. In fact, horses show different selective foraging patterns in comparison with other herbivores [38]: they feed on short grass and are generally monocotyledon specialists, but they expand their diets considerably when food is scarce [54]. In addition, horses cause high trampling pressure. This factor could favor the coverage of trampling-resistant plant species compared to others [38].

The maximum naturalness detected indicated that no alien species were found in the SWEs, either based on their size or treatments. Therefore, in the study area, this suggests that horse grazing did not influence alien plants’ distribution, contrary to previous studies [27]. Finally, contrary to what is reported in previous studies [42,43], no endemic plant species were found in the surveyed areas.

### Implications for Conservation

The SWEs in “Giara di Gesturi” are simultaneously extremely valuable sites for conservation and host priority habitats under the Habitat Directive, and are exposed to pressuring threats such as grazing wild horses. In ecosystems such as SWEs, where several species of conservation interest coexist and interact in a potentially negative way with other species or habitats, the priority interest is to preserve the ecological balance of the system in all its components. In this context, management measures in studied SWEs should reconcile the presence of wild horses and the conservation of their floristic heritage.

The main measure to protect floristic diversity in SWEs should be to exclude the grazing of horses in these areas, or to limit it to certain times of the year. Indeed, fencing had a positive effect on biodiversity in preventing grazing and trampling of plant species by herbivores [31,55], including in ponds [56], although contrasting effects of fencing have been recorded depending on species, in addition to habitat or microhabitat [24]. Therefore, the environmental managers should gather information on SWE systems by analyzing each situation as unique, so that the application of management measures, such as fencing systems, is tailored to each individual case. However, it would not be possible to enclose all the ponds as this would have a negative impact on the wild horse population. In addition, previous studies indicated that grazing by cattle or horses had a positive effect on the number of high-quality habitats’ indicator species, but only in particular ecological conditions (e.g., nutrient-rich areas [57]). Given our findings, following the same methodological approach adopted to protect the endemic plant diversity [58,59], a limited number of small representative areas could be identified and fenced; in fact, it was shown that it is often sufficient to protect small surfaces area to conserve most of the biodiversity present in this territory. Alternatives may include the placement of movable fences, limited to the phenological peak period for the characteristic species of SWEs. This solution would allow controlling (through grazing) the increase in generalist species and the natural evolution of vegetation of SWEs, as suggested by Biggs et al. [56]. The two measures (fixed and mobile fences) could both be contemplated if it is deemed necessary to ensure greater protection of a SWE. These measures, in addition to being relatively simple to implement and maintain, could simultaneously allow the conservation of areas with the highest plant richness and biodiversity, and sustainable management of both the wild horse population and SWEs.

## 4. Materials and Methods

### 4.1. Study Area

The “Giara di Gesturi” is a basaltic plateau, covering a surface of c. 42 km^2^, located in the central-southern part of Sardinia. The average altitude is 550 m a.s.l. with only Mt. Zepparedda (609 m), in the Sothern part, and Mt. Zeppara Manna (580 m), in the northern area, as maximum peaks. The plateau consists of marine sedimentary Miocenic formations overlapped by an alkaline to subalkaline basaltic coverage originated by two different effusive eruptions [60].

The climate shows a typically Mediterranean annual pattern of temperature and precipitation with hot, dry summers and mild, rainy winters; the average temperature is 14.7 °C, with a minimum in January and maximum in July, and the mean rainfall is 741.5 mm per year. Bioclimatically, this area falls within the upper Mesomediterranean phytoclimatic belt, with a subhumid ombrotype.

A typical feature of this area is the presence of several SWEs, locally called *Paùlis*, in which the rainwater persists for many months until late summer. In this area there are *Paùlis* varying in size, most of which having a small surface (*c.* 20 m^2^); others reach 4–5 ha and only three reach 15 ha, and only the latter retain a minimum of water even in the hottest months of summer. This region, due to this uniqueness, hosts peculiar habitats and a characteristic assemblage of flora and fauna, all components of relevant conservation interest. In particular, the *Paùlis* are recognized as particularly important biotopes for many organisms since they host large numbers of species and several rare and threatened animal and plant species [14,61,62,63], strongly contributing to freshwater biodiversity in the Mediterranean region [5]. An additional peculiarity is that the “Giara di Gesturi” hosts the only known population of small wild horses (115–135 cm at the withers); this population consists of about 550 individuals living in small groups. The population size is limited by the extent of the plateau and by the competition for resources and water with other species, especially cattle and goats. As a result of these peculiar ecological, zoological, and botanical features, the “Giara di Gesturi” is recognized as a “Biotopes of national value”, regional natural park and Site of Conservation Interest (SCI code ITB001112) within the Natura 2000 network.

### 4.2. Experimental Design and Data Collection

In order to be able to assume that any differences between the studied plots are the result of the intensity of animal disturbance, this study was conducted in a limited area (the area surrounding the *S’Ala e Mangianu paùli*), with plots located in close proximity to each other, in similar environmental conditions.

Plant diversity was analyzed in the field using non-permanent plots that were randomly placed in the same ecological condition (completely submerged areas with water depth < 10 cm) and at a distance from the edge deemed sufficient to limit the margin effect (Table S3). All plots were surveyed on the same day in June 2021 to reduce the temporal variability in plant turnover. During the sampling activities, a total of 50 plots, each with a size of 0.5 × 0.5 m, were placed; in each plot, the total plant coverage and the relative coverage percentage of each vascular plant were visually estimated. Surveyed plots were equally distributed between small (surface < 5000 m^2^) and large (surface > 5000 m^2^) SWEs. Moreover, each plot was assigned to one level of disturbance following a three-level grid based on the presence and intensity of grazed plants, and the percentage of the total surface affected by animal trampling; the three levels of disturbance identified a priori were: 1—undisturbed plot (no evident or minimal signal of both grazing and trampling; <10% of the plot surface affected); 2—disturbed plot (presence of plant grazed or damaged by animal trampling up to 50% of the plot surface affected by animals); and 3—over-disturbed plot (strongly affected by animal trampling; >50% of the plot surface affected by animals). All the plots clearly affected by the rooting activity of wild boars were excluded from the analysis to avoid an overestimation of the disturbance due to wild horses.

For each plant observed within the plots, the biological, chorological, and ecological types were compiled in a database; the first two categories were mainly obtained from Pignatti [64,65], whereas the ecological type refers to the three categories proposed by Bagella and Caria [43]: specialist of temporary ponds, generalist of wet habitats, and opportunistic terrestrial species. The plant taxonomy was updated following Bartolucci et al. [66]. The complete floristic inventory is reported in Table S1.

### 4.3. Data Analysis

Before starting the analyses, the cover of each plant species visually assessed in the field was transformed using the following scale: 0 (cover = 0%); 1 (cover = 1–25%); 2 (cover = 26–50%); 3 (cover = 51–75%); 4 (cover = 76–100%). The mean scores for each species per size and treatment were summed and reported relative to the maximum possible cover value according to the formula:

Mean Score_ispecies_ = Σscores_ispecies_/no. plots

The PERMANOVA test [67] was used to assess the differences in SWEs’ vegetation by setting SWE size and level of disturbance as variables. The distance between pairs of samples calculated by the Bray–Curtis dissimilarity measure was used on square-root-transformed data for calculating a distance matrix between pairs of samples (i.e., cover values) and significance was assessed through the Monte Carlo test using 999 permutations. *Taxa* responsible for differences among plant assemblages in size and treatment, as indicated by a posteriori testing using PERMANOVA, were identified by similarity percentages (SIMPER) for species contribution analysis. Species contributing more than 10% dissimilarity for any comparison were considered discriminators. The PRIMER statistical package version 7 (Plymouth Routines in Multivariate Ecological Research Statistical Software, v7.0.13, Auckland New Zealand), with the PERMANOVA add-on for multivariate analysis, was used. Finally, principal components analysis (PCA) to visualize and compare the PERMANOVA results was performed using the software BioVinci 2.0. (BioTuring, San Diego, CA, USA).

To support the results obtained in this way, another set of analyses was carried out, mainly based on diversity indices. The effect of SWE size on plant richness was assessed using the non-parametric Kolmogorov–Smirnov test. To measure the overall plant species diversity, considering the natural fragmentation of SWEs, it was assumed that the same approach developed for coastal dunes habitats by Grunewald and Schubert [68], and subsequently implemented by other authors [51,52,53], could be effective. The diversity index (H_modified_, hereafter) was calculated according to the following formula:

H_modified_ = −Σ Pi × ln Pi

where Pi indicates the percentage coverage of each plant.

As in coastal habitats, it was assumed that the H_modified_ value increases as diversity in the community increases [51,52]. Starting from H_modified_, a second index (H_modified-max_, hereafter) was calculated following the formula:

H_modified-max_ = −s × [(ΣPi)/s] × ln [(ΣPi)/s]

where s represents the number of plant species.

To explore the community structure, being independent of species richness, a modified version of the evenness (E_modified_) index was calculated as a ratio between H_modified_ and H_modified-max_. This index ranges from 0 to 1, where 0 indicates that the community is dominated by one species and the other species present diverse but lower abundances, and 1 indicates that all species in the community have the same abundance [51].

The N index was calculated to evaluate the degree of diversity of the native/alien plant species, taking into account the percentage cover of the alien plants along each plot [68]:

N = H_modified_ (without alien plant species)/H_modified_

The N index ranges from 0 to 1, where 0 indicates that the plant diversity is composed exclusively of alien species, and 1 denotes the absence of alien plant species in the plant community [52,68].

Finally, a special focus was made of the endemic component, considered a bioindicator of the good conservation status of a SWE. To do this, the endemicity index proposed by Pinna et al. [52] was calculated according to the formula:

EI = H_modified_ (considering only endemic plant species)/H_modified_

The index ranges from 0 to 1, where 0 indicates the null coverage of endemic plant species, and 1 indicates that the plant cover is composed exclusively of endemic species [52]. The increase in the EI value corresponds to a greater conservation status of SWEs. As proposed in previous studies [51,52,53,68], the Kruskal–Wallis One way Analysis of Variance on Ranks, followed by all pairwise multiple comparison tests, was applied to evaluate significant differences among disturbance levels. Statistical analyses were carried out using the Statistica 8.0 software (Statsoft, Tulsa, OK, USA).

## Figures and Tables

**Table 1:** Mean cover scores for the five discriminant *taxa* in groups of plots sorted by size and treatment. In bold, the highest values recorded for each plant species. Abbreviations: tw = specialist of temporary ponds, gen = generalist of wet habitats, ter = opportunistic terrestrial species. The complete list of mean cover values is reported in Table S2.

	Size		Treatment		
	Small	Large	Undisturbed	Disturbed	Over-Disturbed
*Eudianthe laeta* Rchb. ex Willk. (tw)	**1.76**	0.00	**1.76**	0.00	0.00
*Eleocharis palustris* (L.) Roem. & Schult. subsp. *palustris* (gen)	**1.43**	0.92	**1.41**	1.25	0.25
*Helosciadium crassipes* W.D.J.Koch ex Rchb. (tw)	1.09	**2.46**	1.17	**2.89**	1.00
*Illecebrum verticillatum* L. (ter)	0.13	**0.71**	0.13	**1.00**	0.69
*Alisma plantago-aquatica* L. (gen)	0.00	**0.86**	0.00	0.00	**0.86**

**Table 2:** SIMPER results for discriminant species throughout different size and disturbance levels. Av. Abund: average abundance; Av. Sim.: average Bray–Curtis similarity between all pairs of sites in the group; Sim./SD: ratio of the contribution to the standard deviation; Contrib.%: contribution to the total average similarity; Cum%: cumulative contribution to the total within-group similarity.

	Av. Abund	Av. Sim	Sim/SD	Contrib.%	Cum%
**Small**					
*Eudianthe laeta* Rchb. ex Willk.	32.81	20.56	1.73	47.02	47.02
*Eleocharis palustris* (L.) Roem. & Schult. subsp. *palustris* (gen)	20.80	9.18	0.99	20.99	68.01
*Helosciadium crassipes* W.D.J.Koch ex Rchb.	16.03	5.57	0.71	12.74	80.75
**Large**					
*Alisma plantago-aquatica* L.	6.01	17.61	0.83	37.97	37.97
*Helosciadium crassipes* W.D.J.Koch ex Rchb.	24.80	13.78	0.62	29.72	67.69
*Illecebrum verticillatum* L.	4.02	9.16	0.56	19.76	84.45
**Undisturbed**					
*Eudianthe laeta* Rchb. ex Willk.	32.81	20.56	1.73	47.02	47.02
*Eleocharis palustris* (L.) Roem. & Schult. subsp. *palustris* (gen)	20.80	9.18	0.99	20.99	68.01
*Helosciadium crassipes* W.D.J.Koch ex Rchb.	16.03	5.57	0.71	12.74	80.75
**Disturbed**					
*Helosciadium crassipes* W.D.J.Koch ex Rchb.	59.00	44.39	2.74	77.78	77.78
**Over-disturbed**					
*Alisma plantago-aquatica* L.	10.01	25.15	1.18	60.18	60.18
*Illecebrum verticillatum* L.	6.03	13.09	0.72	31.32	91.50

**Table 3:** Summary of the mean values (±ES) of diversity indices calculated separately based on the SWEs’ size and disturbance level.

	Size		Treatment		
	Small	Large	Undisturbed	Disturbed	Over-Disturbed
**No. species**	14.44 ± 0.33	6.08 ± 0.28	14.44 ± 0.33	6.40 ± 0.37	5.87 ± 0.39
**H_modified_**	1.65 ± 0.07	0.72 ± 0.07	1.65 ± 0.07	0.96 ± 0.09	0.56 ± 0.08
**H_modified-max_**	3.02 ± 0.10	1.15 ± 0.12	3.02 ± 0.10	1.77 ± 0.11	0.74 ± 0.09
**E_modified_**	0.55 ± 0.01	0.68 ± 0.03	0.55 ± 0.01	0.55 ± 0.05	0.76 ± 0.03
**N**	1	1	1	1	1
**EI**	0	0	0	0	0

**Table 4:** Results of non-parametric Kolmogorov–Smirnov test between small and large SWEs.

Variable	Max Neg. Difference	Max. Pos. Difference	*p*-Level	Mean Small	Mean Large	Std. Dev. Small	Std. Dev. Large	Valid N. Small	Valid N. Large
**No. species**	0.0000	1.0000	***p* < 0.001**	14.4400	6.0800	1.6350	1.3820	25	25
**H_modified_**	0.0000	0.8400	***p* < 0.001**	1.6475	0.7223	0.3475	0.3523	25	25
**E_modified_**	−0.6400	0.0400	***p* < 0.001**	0.5464	0.6801	0.0738	0.1591	25	25

**Table 5:** Multiple comparisons z’ values for number of species, H_modified_, and E_modified_ considered separately according to the disturbance level. Significant values are in bold.

		Undisturbed	Disturbed	Over-Disturbed
**No. species** Kruskal–Wallis test: H_(2, N = 50)_ = 37.6312; *p* < 0.0001	**Undisturbed**		**4.363485**	**5.419084**
	**Disturbed**	**4.363485**		0.336067
	**Over-Disturbed**	**5.419084**	0.336067	
**H_modified_** Kruskal–Wallis test: H_(2, N = 50)_ = 35.3240; *p* < 0.0001	**Undisturbed**		**3.135109**	**5.797160**
	**Disturbed**	**3.135109**		1.764353
	**Over-Disturbed**	**5.797160**	1.764353	
**E_modified_** Kruskal–Wallis test: H_(2, N = 50)_ = 25.5510; *p* < 0.0001	**Undisturbed**		0.234675	**4.847770**
	**Disturbed**	0.234675		**3.663133**
	**Over-Disturbed**	**4.847770**	**3.663133**	

## Data Availability

Data are contained within the article or supplementary material.

## Supplementary Materials

The following supporting information can be downloaded at: https://www.mdpi.com/article/10.3390/plants11121597/s1, Table S1: Biological, chorological, and ecological types of the vascular plants surveyed in the SWEs investigated. Abbreviations: tw = specialist of temporary ponds, gen = generalist of wet habitats, ter = opportunistic terrestrial species; Table S2: Mean cover scores for the vascular flora in groups of plots sorted by size and treatment. Abbreviations: tw = specialist of temporary ponds, gen = generalist of wet habitats, ter = opportunistic terrestrial species; Table S3: Location and characteristics of plots in the study area.
