# Supplementary material for: Impact of Horse Grazing on Floristic Diversity in Mediterranean Small Standing-Water Ecosystems (SWEs)
Source: Plants (Basel). 2022 Jun 17;11(12):1597. doi: 10.3390/plants11121597 (PMC9227585; doi:10.3390/plants11121597)
Supplement: Supplementary file 1 [file plants-11-01597-s001.zip › plants-1758866-supplementary.pdf]

plants

MDPI

Supplementary Materials

Table S1. – Biological, chorological, and ecological types of the vascular plants surveyed in the SWEs investigated. Abbreviations: tw= specialist of temporary ponds, gen= generalist of wet habitats, ter= opportunistic terrestrial species.

Taxon	Biological type	Chorological type	Ecological type
Alisma plantago-aquatica L.	I rad	Subcosmop.	gen
Alopecurus bulbosus Gouan subsp. *bulbosus*	H caesp	Euri-Medit.-Subatl.	gen
Baldellia ranunculoides (L.) Parl.	I rad	Medit.-Atlant.	gen
Callitriche brutia Petagna	I rad	Subatlant.	gen
Callitriche stagnalis Scop.	I rad	Eurasiat.	gen
Cynosurus polybracteatus Poir.	T scap	Steno-Medit.-Occid.	ter
Cyperus badius Desf.	G rhiz	Paleo-Subtropic.	gen
Eleocharis palustris (L.) Roem. & Schult. subsp. *palustris*	G rhiz	Subcosmop.	gen
Eryngium corniculatum Lam.	H bienn	Medit.-Occid.	tw
Eudianthe laeta Rchb. ex Willk.	T scap	SW-Medit.	tw
Glyceria notata Chevall.	I rad	Subcosmop.	gen
Helosciadium crassipes W.D.J.Koch ex Rchb.	I rad	Steno-Medit.	tw
Hordeum marinum Huds.	T scap	Euri-Medit.-Occid.	tw
Illecebrum verticillatum L.	T. scap	Sub-Atlant.	ter
Isolepis cernua (Vahl) Roem. & Schult.	T scap	Subcosmop.	tw
Juncus bufonius L.	T caesp	Cosmop.	tw
Juncus effusus L.	H caesp	Cosmop.	gen
Juncus fontanesii J. Gay	G rhiz	Paleo-Subtropic.	gen
Juncus hybridus Brot.	T caesp	Medit.-Atlant.	tw
Juncus pygmaeus Rich. ex Thuill.	T caesp	Medit.-Atlant.	tw
Juncus subnodulosus Schrank	G rhiz	Europ.-Caucas.	gen
Juncus tenageja Ehrh.	T caesp	Paleotemp.	tw
Juncus tingitanus Maire & Weiller	T caesp	Steno-Medit.-Occid.	tw
Kickxia cirrhosa (L.) Fritsch	T scap	Steno-Medit.	tw
Limniris pseudacorus (L.) Fuss	G rhiz	Eurasiat. Temp.	gen
Linum usitatissimum L.	T scap	Coltiv. E subspont.	ter
Lythrum hyssopifolia L.	T scap	Subcosmop.	ter
Mentha pulegium L. *subsp. pulegium*	H scap	Subcosmop.	tw
Myosotis gussoni Jan	T scap	N-Medit. (Euri-)	tw
Myriophyllum verticillatum L.	I rad	Circumbor.	gen
Nasturtium officinale R.Br.	H scap	Cosmop.	gen
Oenanthe silaifolia M.Bieb.	H scap	Medit.-Atlant.	gen
Phalaris coerulescens Desf	H caesp	Steno-Medit.-Macarones	ter
Plantago coronopus L.	T scap	Euri-Medit.	ter
Polypogon maritimus Willd. *subsp. maritimus*	T scap	Steno-Medit.-Macarones	ter
Ranunculus ophioglossifolius Vill.	T scap	Euri-Medit.	tw
Ranunculus paludosus Poir.	H scap	Steno-Medit-.Turan.	ter
Ranunculus sardous Crantz	T scap	Euri-Medit.	gen
Samolus valerandi L.	H scap	Subcosmop.	gen
Teesdalia coronopifolia (J.P. Bergeret) Thell.	T scap	Euri-Medit.	ter
Trifolium resupinatum L.	H rept	(W)-Paleotemp.	ter
Veronica anagalloides Guss.	H scap	Euri-Medit.	tw

Table S2. - Mean cover scores for the vascular flora in groups of plots sorted by size and treatment. Abbreviations: tw= specialist of temporary ponds, gen= generalist of wet habitats, ter= opportunistic terrestrial species.

	SIZE		TREATMENT		
	**Small**	**Large**	**Undisturbed**	**Disturbed**	**Over-disturbed**
Alisma plantago-aquatica L.	0.00	1.00	0.00	0.00	1.00
Alopecurus bulbosus Gouan subsp. *bulbosus*	1.00	0.00	1.00	0.00	0.00
Baldellia ranunculoides (L.) Parl.	1.42	1.17	1.42	1.33	1.00
Callitriche brutia Petagna	1.00	0.00	1.00	0.00	0.00
Callitriche stagnalis Scop.	1.75	0.00	1.75	0.00	0.00
Cynosurus polybracteatus Poir.	0.00	0.00	0.00	0.00	0.00
Cyperus badius Desf.	1.00	1.00	1.00	0.00	1.00
Eleocharis palustris (L.) Roem. & Schult. subsp. *palustris*	1.48	1.33	1.48	1.43	1.00
Eryngium corniculatum Lam.	0.00	0.00	0.00	0.00	0.00
Eudianthe laeta Rchb. ex Willk.	1.91	0.00	1.91	0.00	0.00
Glyceria notata Chevall.	1.40	1.00	1.40	1.00	0.00
Helosciadium crassipes W.D.J.Koch ex Rchb.	1.65	2.42	1.65	2.89	1.00
Hordeum marinum Huds.	0.00	0.00	0.00	0.00	0.00
Illecebrum verticillatum L.	1.00	1.00	1.00	1.00	1.00
Isolepis cernua (Vahl) Roem. & Schult.	1.00	0.00	1.00	0.00	0.00
Juncus bufonius L.	1.00	1.00	1.00	1.00	0.00
Juncus effusus L.	0.00	1.00	0.00	1.00	0.00
Juncus fontanesii J. Gay	0.00	0.00	0.00	0.00	0.00
Juncus hybridus Brot.	0.00	0.00	0.00	0.00	0.00
Juncus pygmaeus Rich. ex Thuill.	1.00	0.00	1.00	0.00	0.00
Juncus subnodulosus Schrank	0.00	0.00	0.00	0.00	0.00
Juncus tenageja Ehrh.	0.00	0.00	0.00	0.00	0.00
Juncus tingitanus Maire & Weiller	1.00	0.00	1.00	0.00	0.00
Kickxia cirrhosa (L.) Fritsch	0.00	0.00	0.00	0.00	0.00
Limniris pseudacorus (L.) Fuss	1.00	1.00	1.00	1.00	0.00
Linum usitatissimum L.	0.00	0.00	0.00	0.00	0.00
Lythrum hyssopifolia L.	0.00	0.00	0.00	0.00	0.00
Mentha pulegium L. subsp. *pulegium*	3.00	1.00	3.00	1.00	1.00
Myosotis gussoni Jan.	0.00	0.00	0.00	0.00	0.00
Myriophyllum verticillatum L.	1.21	1.00	1.21	1.00	0.00
Nasturtium officinale R.Br.	1.33	0.00	1.33	0.00	0.00
Oenanthe silaifolia M.Bieb.	0.00	1.00	0.00	1.00	0.00
Phalaris coerulescens Desf	0.00	0.00	0.00	0.00	0.00
Plantago coronopus L.	0.00	0.00	0.00	0.00	0.00
Polypogon maritimus Willd. subsp. *maritimus*	0.00	0.00	0.00	0.00	0.00
Ranunculus ophioglossifolius Vill.	1.00	0.00	1.00	0.00	0.00
Ranunculus paludosus Poir.	1.00	0.00	1.00	0.00	0.00
Ranunculus sardous Crantz	1.00	1.00	1.00	1.00	0.00
Samolus valerandi L.	0.00	0.00	0.00	0.00	0.00
Teesdalia coronopifolia (J.P. Bergeret) Thell.	0.00	0.00	0.00	0.00	0.00
Trifolium resupinatum L.	0.00	0.00	0.00	0.00	0.00
Veronica anagalloides Guss.	0.00	0.00	0.00	0.00	0.00

Table S3. – Location and characteristics of plots in the study area.

Plot code	SWEs Size	Latitude	Longitude
1	Small	39°45'45.55"N	8°56'47.06"E
2	Small	39°45'45.70"N	8°56'47.47"E
3	Small	39°45'45.77"N	8°56'47.68"E
4	Small	39°45'45.91"N	8°56'47.76"E
5	Small	39°45'46.33"N	8°56'43.92"E
6	Small	39°45'46.68"N	8°56'44.05"E
7	Small	39°45'46.26"N	8°56'46.05"E

8	Small	39°45'46.50"N	8°56'46.78"E
9	Small	39°45'43.30"N	8°57'9.04"E
10	Small	39°45'43.27"N	8°57'9.15"E
11	Small	39°45'43.17"N	8°57'9.22"E
12	Small	39°45'43.09"N	8°57'9.19"E
13	Small	39°45'43.00"N	8°57'9.10"E
14	Small	39°45'42.95"N	8°57'9.03"E
15	Small	39°45'43.09"N	8°57'8.99"E
16	Small	39°45'43.21"N	8°57'8.93"E
17	Small	39°45'44.11"N	8°57'8.68"E
18	Small	39°45'43.97"N	8°57'8.84"E
19	Small	39°45'43.88"N	8°57'8.95"E
20	Small	39°45'44.53"N	8°57'8.28"E
21	Small	39°45'44.57"N	8°57'8.43"E
22	Small	39°45'44.42"N	8°57'8.61"E
23	Small	39°45'43.85"N	8°57'9.40"E
24	Small	39°45'43.82"N	8°57'9.61"E
25	Small	39°45'44.11"N	8°57'9.57"E
26	Large	39°45'40.35"N	8°57'1.02"E
27	Large	39°45'39.80"N	8°57'0.63"E
28	Large	39°45'38.44"N	8°56'59.39"E
29	Large	39°45'38.39"N	8°56'58.55"E
30	Large	39°45'43.42"N	8°56'52.53"E
31	Large	39°45'37.72"N	8°56'58.55"E
32	Large	39°45'43.05"N	8°56'52.29"E
33	Large	39°45'42.69"N	8°56'52.06"E
34	Large	39°45'46.19"N	8°56'55.76"E
35	Large	39°45'47.97"N	8°56'56.51"E
36	Large	39°45'47.64"N	8°56'57.30"E
37	Large	39°45'46.35"N	8°56'58.38"E
38	Large	39°45'45.43"N	8°56'58.55"E
39	Large	39°45'44.30"N	8°56'59.09"E
40	Large	39°45'43.52"N	8°57'0.70"E
41	Large	39°45'42.55"N	8°57'1.62"E
42	Large	39°45'41.51"N	8°57'1.76"E
43	Large	39°45'36.60"N	8°56'58.09"E
44	Large	39°45'35.96"N	8°56'57.76"E
45	Large	39°45'34.90"N	8°56'56.48"E
46	Large	39°45'34.85"N	8°56'56.25"E
47	Large	39°45'36.05"N	8°56'54.76"E
48	Large	39°45'37.26"N	8°56'53.73"E
49	Large	39°45'39.33"N	8°56'52.88"E
50	Large	39°45'39.65"N	8°56'52.84"E
